# Obtaining a free vertical margin is challenging in endoscopic submucosal dissection of a rectal neuroendocrine tumor: use of adaptive traction to improve exposure in a child

**DOI:** 10.1055/a-2085-0449

**Published:** 2023-05-26

**Authors:** Timothée Wallenhorst, Louis-Jean Masgnaux, Jean Grimaldi, Romain Legros, Jérôme Rivory, Jérémie Jacques, Mathieu Pioche

**Affiliations:** 1 Gastroenterology and Endoscopy Unit, Pontchaillou University Hospital, Rennes, France; 2 Gastroenterology and Endoscopy Unit, Edouard Herriot Hospital, Hospices Civils de Lyon, Lyon, France; 3 Gastroenterology and Endoscopy Unit, Dupuytren University Hospital, Limoges, France

Both European Society of Gastrointestinal Endoscopy (ESGE) and American guidelines suggest resection of small rectal neuroendocrine tumors (NETs) by endoscopic submucosal dissection (ESD) for lesions up to 20 mm 1 2 . A completely resected rectal NET (R0) with no pejorative factors for recurrence (< 10 mm, T1, grade 1, no lymphovascular invasion) requires no additional exploration and no follow-up 3 .

Obtaining healthy horizontal margins is easy, but it is sometimes more difficult to obtain free vertical margins above the point of deepest submucosal invasion. For this, it is necessary to perform a deep submucosal dissection, sliding along the muscular layer; strong and adaptive traction seems to us to be very useful to facilitate this. We describe a new traction device, the A-TRACT-2 4 , that allows the strength of traction to be adapted to improve the exposure of the deep submucosa.

We report here a never previously documented case of a 13-year-old child with a 7-mm rectal NET ( Video 1 ). After complete circumferential incision and trimming, we used clips to set up the traction device on both poles of the lesion ( Fig. 1 ). We then fixed the rubber band to the opposite wall of the lumen, creating the initial traction. When we noticed that the traction was weaker during the submucosal dissection, we tightened the device by pulling on the loop, bringing the two anchoring points of the device closer both to each other and to the rubber band in order to renew the optimal level of traction. The submucosal exposure was ideal right through until the end of the procedure, leading to a curative R0 resection of a 7-mm grade 1 NET, with large free vertical margins.

**Video 1** Endoscopic resection of a rectal neuroendocrine tumor (NET) in a child using an adaptive traction strategy.


**Fig. 1:**
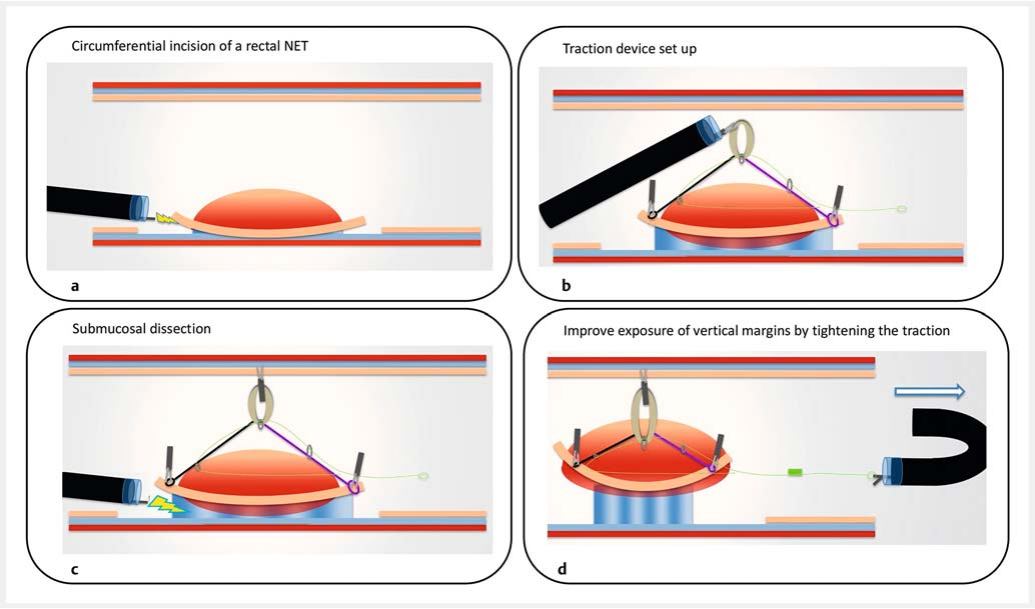
Schematic representation of submucosal dissection for a rectal neuroendocrine tumor (NET) using an adaptive traction system (the A-TRACT-2 device) showing: **a** circumferential incision of the rectal NET; **b** the traction device set up; **c** submucosal dissection with traction applied; **d** improved exposure of the vertical margins achieved by tightening of the traction device.

This technique seems attractive, especially for the resection of NETs, where strong adjustable traction is useful to be sure of achieving R0 vertical margins.

Endoscopy_UCTN_Code_TTT_1AQ_2AD

## Notes

Endoscopy E-Videos

https://eref.thieme.de/e-videos
*E-Videos* is an open access online section of the journal *Endoscopy* , reporting on interesting cases and new techniques in gastroenterological endoscopy. All papers include a high-quality video and are published with a Creative Commons CC-BY license. Endoscopy E-Videos qualify for HINARI discounts and waivers and eligibility is automatically checked during the submission process. We grant 100% waivers to articles whose corresponding authors are based in Group A countries and 50% waivers to those who are based in Group B countries as classified by Research4Life (see: https://www.research4life.org/access/eligibility/ ). This section has its own submission website at https://mc.manuscriptcentral.com/e-videos

## References

[1] Deprez P H Moons L MG OʼToole D Endoscopic management of subepithelial lesions including neuroendocrine neoplasms: European Society of Gastrointestinal Endoscopy (ESGE) Guideline Endoscopy 2022 54 412 429 35180797 10.1055/a-1751-5742

[2] Fine C Roquin G Terrebonne E Endoscopic management of 345 small rectal neuroendocrine tumours: A national study from the French group of endocrine tumours (GTE) United European Gastroenterol J 2019 7 1102 1112 10.1177/2050640619861883 PMC6794692 31662867

[3] de Mestier L Lepage C Baudin E Thésaurus National de Cancérologie Digestive (TNCD). Digestive Neuroendocrine Neoplasms (NEN): French Intergroup clinical practice guidelines for diagnosis, treatment and follow-up (SNFGE, GTE, RENATEN, TENPATH, FFCD, GERCOR, UNICANCER, SFCD, SFED, SFRO, SFR) Dig Liver Dis 2020 52 473 492 32234416 10.1016/j.dld.2020.02.011

[4] Masgnaux L J Grimaldi J Legros R Endoscopic submucosal dissection in the colon using a novel adjustable traction device: A-TRACT-2 Endoscopy 2022 54 02 E988 E989 35926531 10.1055/a-1888-3963 PMC9736814

